# Mapping geographic clusters of new HIV diagnoses to inform granular-level interventions for HIV epidemic control in western Kenya

**DOI:** 10.1186/s12889-021-11890-7

**Published:** 2021-10-23

**Authors:** Hellen Muttai, Bernard Guyah, Thomas Achia, Paul Musingila, Jesse Nakhumwa, Rose Oyoo, Wilfrida Olweny, Redempter Odeny, Spala Ohaga, Kawango Agot, Kennedy Oruenjo, Bob Awino, Rachael H. Joseph, Fredrick Miruka, Emily Zielinski-Gutierrez

**Affiliations:** 1 Division of Global HIV & TB, United States Centers for Disease Control and Prevention, 00621 Nairobi, Kenya; 2 School of Public Health, Maseno University, Kisumu, Kenya; 3 Impact Research and Development Organization, Kisumu, Kenya; 4 Siaya County Department of Health, Siaya, Kenya; 5 United States Centers for Disease Control and Prevention, Guatemala, Guatemala

**Keywords:** Geospatial analysis, Mapping geographic clusters, New HIV diagnoses, Kenya

## Abstract

**Background:**

As countries make progress towards HIV epidemic control, there is increasing need to identify finer geographic areas to target HIV interventions. We mapped geographic clusters of new HIV diagnoses, and described factors associated with HIV-positive diagnosis, in order to inform targeting of HIV interventions to finer geographic areas and sub-populations.

**Methods:**

We analyzed data for clients aged > 15 years who received home-based HIV testing as part of a routine public health program between May 2016 and July 2017 in Siaya County, western Kenya. Geospatial analysis using Kulldorff’s spatial scan statistic was used to detect geographic clusters (radius < 5 kilometers) of new HIV diagnoses. Factors associated with new HIV diagnosis were assessed in a spatially-integrated Bayesian hierarchical model.

**Results:**

Of 268,153 clients with HIV test results, 2906 (1.1%) were diagnosed HIV-positive. We found spatial variation in the distribution of new HIV diagnoses, and identified nine clusters in which the number of new HIV diagnoses was significantly (1.56 to 2.64 times) higher than expected. Sub-populations with significantly higher HIV-positive yield identified in the multivariable spatially-integrated Bayesian model included: clients aged 20–24 years [adjusted relative risk (aRR) 3.45, 95% Bayesian Credible Intervals (CI) 2.85–4.20], 25–35 years (aRR 4.76, 95% CI 3.92–5.81) and > 35 years (aRR 2.44, 95% CI 1.99–3.00); those in polygamous marriage (aRR 1.84, 95% CI 1.55–2.16), or separated/divorced (aRR 3.36, 95% CI 2.72–4.08); and clients who reported having never been tested for HIV (aRR 2.35, 95% CI 2.02–2.72), or having been tested > 12 months ago (aRR 1.53, 95% CI 1.41–1.66).

**Conclusion:**

Our study used routine public health program data to identify granular geographic clusters of higher new HIV diagnoses, and sub-populations with higher HIV-positive yield in the setting of a generalized HIV epidemic. In order to target HIV testing and prevention interventions to finer granular geographic areas for maximal epidemiologic impact, integrating geospatial analysis into routine public health programs can be useful.

**Supplementary Information:**

The online version contains supplementary material available at 10.1186/s12889-021-11890-7.

## Background

In 2017, the eastern and southern Africa region had an estimated 800,000 new HIV infections, accounting for 44% of all new infections worldwide [1]. Reducing new HIV infections is essential for HIV epidemic control [2]. Interventions to prevent transmission of HIV include biomedical (e.g. antiretroviral drug use, medical male circumcision, condom use), behavioral (reducing risky behaviors) and structural (policy formulation and guidelines). Initiation of antiretroviral therapy (ART) at, or soon after, HIV diagnosis, and sustained viral suppression, substantially reduces HIV transmission [3, 4] and HIV-related morbidity and mortality [5]. In 2014, the Joint United Nations Programme on HIV/AIDS (UNAIDS) set ambitious global targets towards achieving HIV epidemic control, recommending programs aim for 90% of people living with HIV (PLHIV) to know their HIV status, 90% of people with diagnosed HIV infection to receive sustained ART, and 90% of people receiving ART to achieve viral suppression [6].

In 2017, Kenya had an estimated HIV prevalence of 4.8% among individuals aged 15–49 years, and approximately 52,800 new HIV infections. Nationally, with 1.12 million of the estimated 1.5 million PLHIV accessing ART, the country had achieved a population ART coverage of 75% [7]. Of Kenya’s 47 counties, Siaya had the highest HIV prevalence of 21%, with an estimated 123,000 PLHIV, and 4000 new HIV infections [7]. As of September 2017, the county had a population ART coverage of 71% [7]. In order to accelerate progress towards HIV epidemic control, programs in Siaya intensified implementation of multiple county-wide HIV prevention interventions and testing approaches, including community home-based HIV testing.

In many countries with generalized HIV epidemics, interventions and resource allocation are planned and targeted to large geographic units (primarily county-level in Kenya). As more PLHIV access HIV services, interventions need to reach a diminishing number of people. Strategies to effectively target the delivery of HIV services, including HIV testing, to finer geographic units and sub-populations, are therefore needed, to improve program efficiency.

Geospatial analysis and mapping have been used to demonstrate geospatial clustering (i.e. “micro-epidemics”) of HIV infection around geographic, social or behavioral risk factors [8–11]; describe geographic clustering of incident HIV infections [12]; demonstrate within-country variability in the decline of HIV prevalence [13]; describe geographic variability in prevention of mother to child transmission program achievements [14] and variability in HIV testing access [15]; and to guide targeted implementation of HIV testing interventions [16, 17]. Furthermore, multiple studies have described ways to prioritize HIV interventions to specific geographic areas, including areas with higher HIV prevalence [11, 18], higher HIV incidence [18, 19], and focused prioritization based on local epidemiologic context [20]. Despite the potential utility of geospatial analysis of routine HIV testing program data to map finer geographic clusters of higher new HIV diagnoses in order to inform targeting of HIV testing and prevention interventions, limited studies have been conducted on this. One study done in Kenya used 2015/2016 facility-level HIV testing data to describe the spatial distribution of newly diagnosed HIV-positive persons across counties with differing HIV burden [21].

This study uses data from home-based HIV testing conducted as part of a routine public health program in Siaya County, western Kenya. We use geospatial analysis to assess and map granular geographic clusters of new HIV diagnoses, and a spatially-integrated Bayesian hierarchical model to describe factors associated with new HIV diagnoses in order to inform targeting of HIV interventions to finer geographic areas and sub-populations.

## Methods

### Study area, design and setting

This study uses data from home-based HIV testing offered as part of a routine public health program in Siaya County. Home-based testing was supported by the United States President’s Emergency Plan for AIDS Relief (PEPFAR) through the United States Centers for Disease Control and Prevention (CDC), under the Impact Research and Development Organization cooperative agreement.

Siaya County borders Lake Victoria in western Kenya. The population is predominantly rural, and includes fishing communities living along the lake’s beaches. Administratively, the county consists of six sub-counties, which are subdivided into 30 wards, and further into 179 sub-locations, and 2285 villages. In 2016 and 2017 intensified routine HIV testing was implemented in Siaya County, and included biannual testing offered to fishing communities living along the beaches, and home-based testing offered to inland residents of the county.

For home-based HIV testing, all households in the inland geographic areas were visited to enumerate occupants and assess their eligibility for HIV testing. Household occupants were enumerated if they would be resident in the household for one or more months following enumeration. Clients aged > 15 years were eligible for HIV testing if they reported having never been tested for HIV; reported a negative HIV test done more than 3 months ago; had signs, symptoms or a diagnosis of tuberculosis, or a sexually transmitted infection; or reported a recent (within 3 months) HIV exposure such as unprotected sex with a partner of unknown or positive HIV status. Children aged 14 years and below were eligible for testing if their biological mother was known to be HIV infected or deceased. Within 1 month of enumeration, trained lay counselors offered pre-test counseling, HIV testing and post-test counseling to those eligible. Counselors made up to three follow-up visits to offer testing to those not found at home. HIV testing was offered according to the 2015 Kenya HIV testing guidelines [22] using Determine™ [23] and First Response® [24] rapid point of care kits.^1^ An individual was considered HIV-negative (uninfected) if the Determine test result was negative (considered a conclusive negative result), HIV-positive (infected) if both the Determine and First Response serial tests results were positive (considered a conclusive positive result), and inconclusive if the Determine test was positive and First Response test was negative. Clients with inconclusive HIV test results were referred to a health facility for follow-up testing according to Kenya Ministry of Health guidelines.

We retrospectively analyzed data for clients aged > 15 years who received routine home-based HIV testing in Siaya County from May 2016 to July 2017. Home-based testing data for children aged < 15 years, and data collected as part of biannual HIV testing of fishing communities were excluded from the analysis. Data were spatially analyzed at sub-location level; sub-locations in which all, or more than half of households were enumerated, were included in the analysis. Out of the 179 sub-locations in the county, data from 161 sub-locations met criteria for inclusion (156 sub-locations in which all households were enumerated, and 5 in which > 50% of households were enumerated).

### Data management

Routine home-based HIV testing data collected included sociodemographic characteristics: age, sex, marital status and relationship to household head; sub-county, ward, sub-location and village of residence; and HIV test eligibility criteria and test results. Data collected were manually recorded on standardized enumeration forms and Ministry of Health HIV testing registers by lay counselors. At a central (office) location, data clerks reviewed the data for completeness and accuracy, and entered it into a secure password-protected Microsoft Access database.

For this study, routinely collected data were stripped all identifiers (names and unique patient numbers) and each record assigned a new study-specific identification number. The analytic dataset was saved in a secure password-protected database.

### Data analysis

Frequencies, proportions, medians and interquartile ranges were calculated to summarize the data. The proportion of new HIV-positive clients (new HIV-positive yield) was defined as the total number of clients newly identified HIV-positive among those with a conclusive test result. The proportion of total HIV-positive clients was calculated as the sum of new HIV-positive and previously-identified HIV-infected clients among those assessed for HIV test eligibility.

### Spatial data analysis

For spatial analysis, client data were aggregated to the sub-location where they were tested for HIV, and sub-location-level geographic units used for analysis and mapping. Village-level analysis was not possible owing to small numbers and lack of household-level point coordinates.

#### a) Global Moran’s I statistic

The Global Moran’s I statistic was computed using GeoDa software tool version 1.12.1.131 [25, 26] in order to assess the presence of spatial autocorrelation of new HIV diagnoses at sub-location level. A significant positive autocorrelation indicates the existence of either high-value or low-value clustering, while a negative autocorrelation indicates a tendency toward the juxtaposition of high values next to low values.

#### b) Kulldorff’s spatial scan statistic

The Kulldorff’s spatial scan statistic [27] was implemented using SaTScan™ version 9.6 [28] to detect spatial clusters of new HIV diagnoses. Since the proportion of clients newly diagnosed HIV-positive was low, a discrete Poisson probability model was used for scanning. SaTScan™ software cyclically scans a window across space, calculating the number of observed and expected cases inside the window at each location, and adjusting for spatial inhomogeneity of the background population. The window with the maximum likelihood estimate is considered to be the most likely cluster, rejecting the null hypothesis of no clusters at *p* value < 0.05. For our study, the Kulldorff spatial cluster detection looped over all of the 161 sub-locations included in the analysis. We used a maximum spatial cluster size radius of five kilometers (km) in order to inform HIV program implementation meaningfully at a granular sub-location level. Because Siaya County has a generalized epidemic, and it was not possible to segregate the population proportion at higher risk, we assumed 50% of the total population were at risk of HIV-infection (excluding PLHIV with previously known HIV status) [29]. The maximum number of standard Monte Carlo replications was set to 999. Significant clusters were reported together with corresponding radii, number of observed and expected cases, relative risk, likelihood ratio and *p*-values. Clusters with a relative risk of > 1.0 at *p* value < 0.05 were considered significant clusters of higher new HIV diagnoses; while those with a relative risk of < 1.0 at *p* value < 0.05 were considered significant clusters of lower new HIV diagnoses. A standard Geographical Information System (GIS) program, Quantum GIS version 3.6 [30], was used to map clusters and layer them over ecological features.

#### c) Mapping of HIV testing uptake

To describe patterns of HIV testing uptake, quantiles of testing uptake were mapped and overlaid on sub-location clusters of new HIV diagnoses.

#### d) Bayesian hierarchical spatial model

We used a Bayesian hierarchical spatial model to assess the relationship between new HIV diagnosis and covariates while accounting for spatial autocorrelation in the data. A Bayesian estimation based on an Integrated Nested Laplace approximation (INLA) was computed using R-INLA package [31]. In a Bayesian framework random effects are unknown quantities assigned to prior distributions that reflect prior knowledge on the structure of the effects, while enabling accounting for heterogeneity across spatial units. We applied a Bayesian approach to client-level and spatial parameters, separately and jointly.

The outcome in our analysis was new HIV-positive diagnosis. The covariates: age, sex, marital status, time since last HIV test and sub-location proportion of total HIV-positive clients, were included in the Bayesian spatial model.

We let *Y*_*ijklm*_ denote the number of new HIV-positive individuals diagnosed among the *n*_*ijklm*_ tested for HIV in the *i*-th sub-location for the *j*-th age category, *k*-th sex, *l*-th marital status and *m*-th time since last HIV test. We assumed that *Y*_*ijklm*_ is a Poisson random variable with mean *E*_*ijklm*_*θ*_*ijklm*_. That is, *Y*_*ijklm*_~*Poisson*(*E*_*ijklm*_*θ*_*ijklm*_), where *E*_*ijklm*_ denotes the expected number of cases and *θ*_*ijklm*_ is the “true” but unknown relative risk in the *i*-th sub-location for the *j*-th age category, *k*-th sex, *l*-th marital status and *m*-th time since last HIV test.

We used the Besag-York-Mollié (BYM) model [32, 33] of the form:

$$ \log \left({\pi}_{ijklm}\right)={\beta}_0+{\boldsymbol{X}}_{ijklm}\boldsymbol{\beta} +{u}_i+{v}_i $$

where *β*_0_ is the intercept that represents the overall log-odds of a new HIV-positive diagnosis; ***β*** is a vector of parameters associated with the vector of covariate ***X***_*ijklm*_; *u*_*i*_ is a spatial structured component modeled with a conditional autoregressive (CAR) distribution ${u}_i\mid {\boldsymbol{u}}_{-i}\sim N\left({\overline{u}}_{\delta_i},\frac{\sigma_u^2}{n_{\delta_i}}\right),$ where ${\overline{u}}_{\delta_i}={n_{\delta_i}}^{-1}\sum \limits_{j\in {\delta}_i}{u}_{j,}$ *δ*_*i*_ and ${n}_{\delta_i}$ represent the set of neighbors and the number of neighbors of sublocation *i* respectively; and *v*_*i*_ is an unstructured spatial effect defined as ${v}_i\sim N\left(0,{\sigma}_v^2\right)$. The Besag York Mollié Poisson model [32] includes an ordinary random-effects component for non-spatial heterogeneity.

The posterior distributions of the parameters in the Bayesian spatial model were estimated via an Integrated Nested Laplace Approximation (INLA) approach in R statistical package, borrowing strength across sublocations to produce smoothed sublocation level estimates even where the data were sparse. Full list of the latent models, likelihoods and prior assumptions can be found in the R-INLA website at http://www.r-inla.org/ [31].

Unadjusted relative risk (uRR) and 95% Bayesian credible intervals (CIs) were computed to describe univariate associations. A multivariable Bayesian spatial Poisson model was used to assess the performance of four non-spatial and spatial models: fixed effects only, fixed effects in a spatially unstructured model, fixed effects in a spatially structured model, and fixed effects in a convolution unstructured and structured spatial random effects model. The convolution model, additionally allows for both spatially structured and unstructured heterogeneity in one model [34]. We reported measures of adjusted relative risk (aRR), 95% Bayesian CIs, precision of the spatially unstructured and structured random effect model, and the deviance information criterion (smaller values indicating better model performance).

Random effects maps of residual variability of new HIV diagnoses, not accounted for by the explanatory variables, were generated from the convolution Bayesian Poisson model, and mapped using ggplot2 R package [35]. These included unstructured random effects maps, showing variability when spatial autocorrelation was not taken into account, and structured random effects maps, when spatial autocorrelation was accounted for.

The Bayesian approach allows the posterior probability of any area’s relative risk exceeding a threshold to be calculated. A threshold of 1.25 was used in our analysis; noting that it would have been possible to use a different threshold > 1.0 (denoting an area’s higher relative risk). This probability is an important tool for the assessment of unusual elevated risk of disease [36, 37]. From the posterior marginals of the relative risk, ${\hat{\theta}}_{ijklm}=\exp \left({\hat{\beta}}_0+{\boldsymbol{X}}_{ijklm}\hat{\boldsymbol{\beta}}+{u}_i+{v}_i\right),$ the exceedance probability was calculated and is defined as $\Pr \left(\theta >{\theta}^{\ast}\right)={\sum}_{g=1}^GI\left({\theta}^{(g)}>{\theta}^{\ast}\right),$ where G is the sampler sample size. Wherever this probability is high there is evidence that the excess risk is not only high, but significantly high.

## Results

**Fig. 1:**
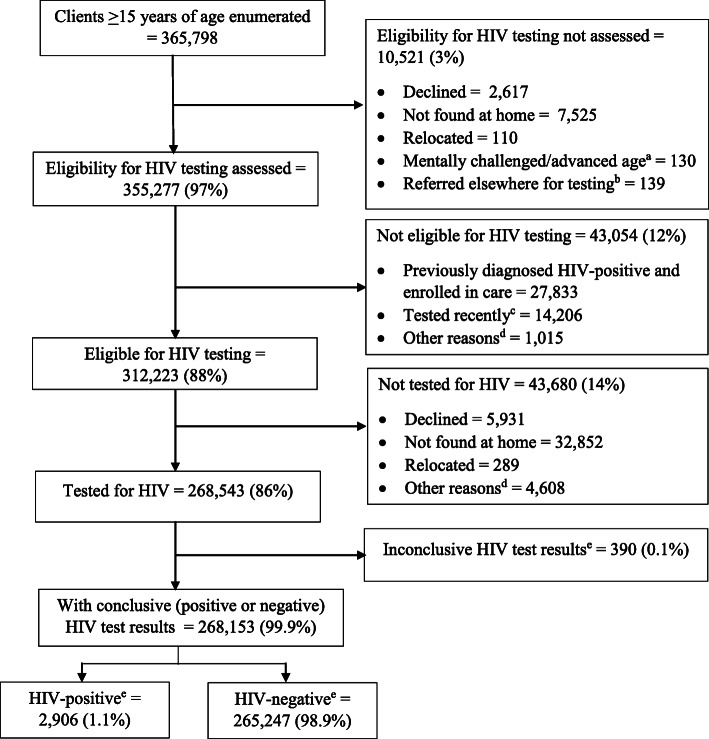
Flowchart of clients receiving home-based HIV testing, Siaya County, May 2016–July 2017*.* ^a^Advanced age referred to elderly clients who were unable to comprehend HIV testing due to their diminished mental capacity related to old age. ^b^Clients 15–24 years of age in selected sub-locations were referred to another program offering testing for young people. ^c^Self-reported tested recently within the prior 3 months. ^d^Details of other reasons not given. ^e^An individual was considered HIV-negative (uninfected) if the Determine test result was negative (considered a conclusive negative result), HIV-positive (infected) if both the Determine and First Response serial tests results were positive (considered a conclusive positive result), and inconclusive if the Determine test was positive and First Response test was negative

From the 161 Siaya administrative sub-locations included in the analysis, 365,798 clients aged > 15 years from 136,607 households were enumerated for home-based HIV testing (Fig. 1). Among those enumerated, 136,607 (37%) were household-heads, 80,161 (22%) were spouses, 110,255 (30%) were children aged > 15 years, and 38,775 (11%) were other relatives/non-relatives (Table 1). Overall, those enumerated had a median age of 30 years (interquartile range 20–47 years), and 203,170 (56%) were women.

**Table 1:** Characteristics of clients aged > 15 years offered home-based HIV testing in Siaya County

	Enumerated, n (%)	Eligibility assessed, n (%)	Eligible for HIV testing, n (%)	Tested for HIV, n (%)	With conclusive test results, n	HIV-positive, n (%)
Total clients	365,798 (100%)	355,277 (97%)	312,223 (88%)	268,543 (86%)	268,153	2906 (1.1%)^a^
Relationship to household head						
Household head^b^	136,607 (37%)	132,622 (37%)	111,024 (36%)	94,506 (35%)	94,349	1432 (1.5%)
Spouse	80,161 (22%)	78,756 (22%)	66,124 (21%)	60,660 (23%)	60,545	848 (1.4%)
Children > 15 years	110,255 (30%)	105,999 (30%)	99,144 (32%)	80,781 (30%)	80,698	345 (0.4%)
Relatives & non-relatives	38,775 (11%)	37,900 (11)	35,931 (11%)	32,596 (12%)	32,561	281 (0.9%)
Age (median, interquartile range)	30 (20, 47)	30 (20, 47)	28 (19, 46)	28 (19, 47)		
Age group (years)						
15–19	88,758 (24%)	85,813 (24%)	81,979 (26%)	69,651 (26%)	69,580	166 (0.2%)
20–24	52,952 (14%)	51,579 (14%)	47,722 (15%)	41,738 (16%)	41,682	442 (1.1%)
25–35	82,771 (23%)	80,349 (23%)	67,381 (22%)	57,238 (21%)	57,138	1096 (1.9%)
> 35	141,317 (39%)	137,536 (39%)	115,141 (37%)	99,916 (37%)	99,753	1202 (1.2%)
Sex						
Men	162,628 (44%)	156,410 (44%)	141,011 (45%)	114,349 (43%)	114,187	1123 (1.0%)
Women	203,170 (56%)	198,867 (56%)	171,212 (55%)	154,194 (57%)	153,966	1783 (1.2%)
Marital status^c^						
Single				102,988 (38%)	102,887	442 (0.4%)
Married monogamous				131,034 (49%)	130,802	1844 (1.4%)
Married polygamous				6284 (2%)	6275	154 (2.5%)
Separated/divorced				1917 (1%)	1913	100 (5.2%)
Widow/widower				26,317 (10%)	26,273	363 (1.4%)
Time since last HIV test^c^						
< 3 months				2521 (1%)	2516	32 (1.3%)
3–12 months				183,854 (69%)	183,606	1711 (0.9%)
> 12 months				64,870 (24%)	64,761	951 (1.5%)
Never tested				17,298 (6%)	17,270	212 (1.2%)

^a^In addition to the new diagnoses of 2906, a total of 27,833 previously diagnosed HIV-positive clients were identified; the proportion of total HIV-positive clients was 8.7% among those whose eligibility was assessed

^b^Among household heads, 81,599 (60%) were men and 55,008 (40%) women

^c^These variables were collected only for clients tested for HIV

Abbreviation: n, number

Of the total clients enumerated, 355,277 (97%) were assessed for HIV testing eligibility, and 312,223 (88%) were eligible for testing (Fig. 1, Table 1). Among those eligible, 268,543 (86%) were tested for HIV, and 2906 (1.1%) of 268,153 clients with conclusive HIV test results were diagnosed HIV-positive. The new HIV positive yield by different characteristics is shown in Table 1 and supplemental Fig. S1. The reasons for not testing among eligible clients are shown in Fig. 1.

The 161 sub-locations had a median HIV testing uptake among eligible clients of 87% (interquartile range 82–91%), a median new HIV-positive yield of 1.1% (interquartile range 0.8–1.5%), and a median proportion of total HIV-positive clients of 9.1% (interquartile range 7.6–10.4%), (Table 1). Maps showing the sub-location distribution of new HIV-positive yield, proportion of total HIV-positive clients, and the distribution of different client characteristics are shown in supplemental Fig. S2.

### Spatial clusters of new HIV diagnoses

**Fig. 2:**
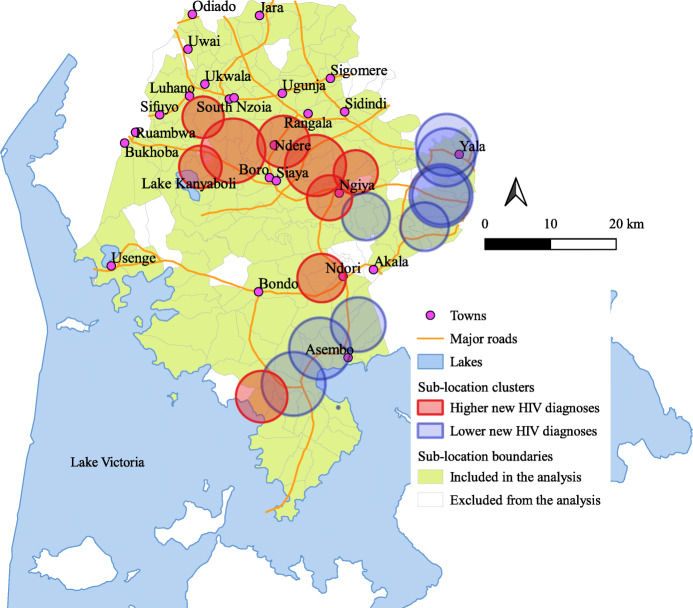
Sub-location clusters of new HIV diagnoses from home-based HIV testing in Siaya County. Spatial clusters of new HIV diagnoses were detected using the Kulldorff’s spatial scan statistic, implemented using SaTScan™ version 9.6 (http://www.satscan.org). Spatial clusters were mapped and layered over ecological features using a standard Geographical Information System (GIS) program, Quantum GIS version 3.6 (http://qgis.org). Shown in red circles are sub-location clusters of higher new HIV diagnoses with a relative risk of > 1.0 at *p* value< 0.05, and in blue circles sub-location clusters of lower new HIV diagnoses with a relative risk of < 1.0 at *p* value< 0.05. The boundary information for sub-locations in Siaya was obtained as shapefiles from DIVA-GIS (https://www.diva-gis.org/gdata)

Sub-location level Moran’s I analysis yielded an index of 0.2925 (*p* value < 0.001), indicating the presence of significant spatial autocorrelation of new HIV diagnoses. Nine significant sub-location clusters of higher new HIV diagnoses were identified (Fig. 2, Table 2) with cluster relative risk ranging from 1.56 to 2.64, and radius ranging from 3.15 to 4.91 km. Seven of the nine clusters were located centrally in the area around, and stretching eastward and westward of Ndere town; one cluster was in the area around Ndori town, where four major roads intersect; and another was located in the south, adjacent to Lake Victoria (Fig. 2). The sub-location cluster with the highest relative risk of 2.64 was located north-east of Ngiya town in a predominantly rural area. Significant clusters of lower new HIV diagnoses were located in the south-eastern part of the county (Fig. 2, Table 2), the area around and stretching southward of Yala town; the area south-east of Ngiya town; and the area adjacent to Lake Victoria, and stretching north, west and south-west of Asembo town. Major roads passed through areas with clusters of higher and lower new HIV diagnoses.

**Table 2:** Characteristics of clusters of new HIV diagnosesa in Siaya County

Number of sub-locations in the cluster	Names of sub-locations in the cluster	Radius (kilometers)	Observed cases	Expected cases	Relative risk	Log likelihood ratio	***P*** value
**Clusters with significant (*p*** **value < 0.05) higher new HIV diagnoses**							
3	Malunga West, Sirembe, Malunga East	3.36	49	18.79	2.64	16.91	< 0.001
2	Gangu, Ojwando ‘A’	3.24	62	28.57	2.2	14.81	< 0.001
7	Kochieng ‘A’, Kodiere, Ojwado ‘B’, Kochieng ‘B’, Koyeyo, Komeny, Kalaka, Ojwando ‘A’	4.91	145	70.32	2.12	31.24	< 0.001
5	Komolo, Hono, Kukumu_kombewa, Nyalgunga, Koyeyo	3.95	140	72.96	1.97	25.01	< 0.001
4	Komenya Kowala, Kalkada Uradi, Komenya Kalaka, Simur Kondiek	3.15	72	38.93	1.87	11.4	0.002
7	Ulafu, Umala, Nyalgunga, Nyamila, Olwa, Hono, Karapul	4.65	197	111.58	1.82	27.89	< 0.001
4	Mur_ngiya, Olwa, Masumbi, Umala	3.43	91	57.76	1.59	8.32	0.026
3	Bar Chando, Abom, North Ramba	3.69	97	62.91	1.56	8.12	0.032
2	Kagwa, Kokwiri	3.92	81	47.93	1.71	9.62	0.008
**Clusters with significant (*p*** **value < 0.05) lower new HIV diagnoses**							
5	Gombe, Onyinyore, Ramula, Kambare, Uranga	3.69	68	115.55	0.58	11.9	< 0.001
5	Omia Malo, Omia Diere, Memba, South Ramba, Omia Mwalo	4.14	81	150.33	0.53	20.11	< 0.001
4	Lihanda, Uranga, Marenyo, Ramula	4.38	78	146.24	0.52	20.05	< 0.001
6	Bar Sauri, Nyamninia, Anyiko_yala, Jina, Nyawara, Nyandiwa_yala	4.71	80	154.24	0.51	22.72	< 0.001
5	Dienya East, Nguge, Dienya West, Ulamba, Wagai West	3.61	32	62.12	0.51	9.05	0.014
7	Nyamninia, Bar Sauri, Jina, Nyandiwa_yala, Anyiko_yala, Nyawara, Marenyo	4.41	99	192.71	0.5	29.37	< 0.001
5	Lihanda, Uranga, Marenyo, Ramula, Nyandiwa_yala	4.78	86	180.17	0.46	32.17	< 0.001
4	Mahaya, Akom, Memba, Nyagoko	4.68	56	119.77	0.46	21.92	< 0.001
5	Masala, Rachar, Akom, Kobong’, Nyagoko	4.85	63	164.62	0.37	42.97	< 0.001
1	Ochieng’a	0	2	31.7	0.06	24.33	< 0.001

^a^Sub-location clusters of new HIV diagnoses were mapped using SaTScan, which gradually scans a window cyclically across space, noting the number of observed and expected observations inside the window at each location, adjusting for the underlying spatial inhomogeneity of the background population

### Sub-location patterns of HIV testing uptake

**Fig. 3:**
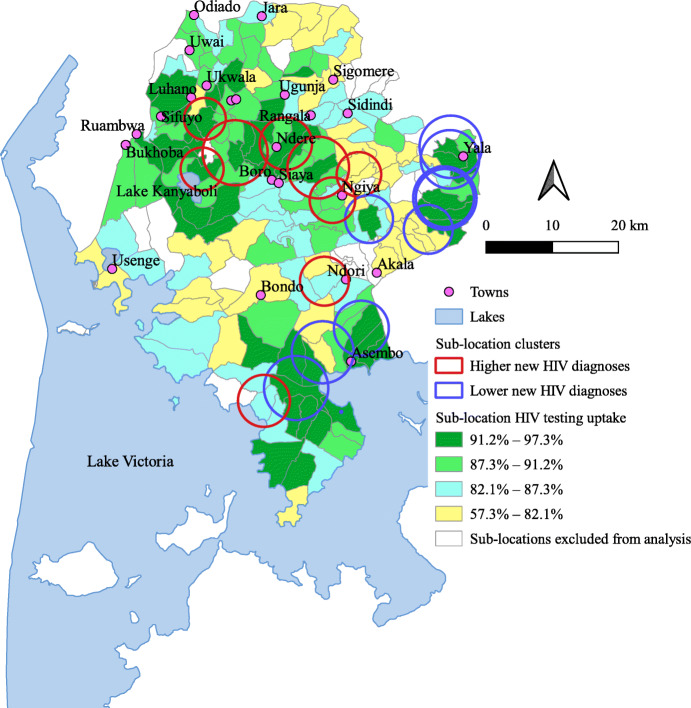
Sub-location HIV testing uptake overlaid with clusters of new HIV diagnoses, Siaya County. A standard Geographical Information System (GIS) program, Quantum GIS version 3.6 (http://qgis.org) was used to map HIV testing uptake, and overlay sub-location clusters of new HIV diagnoses. The sub-location HIV testing uptake is in quantiles. The clusters of new HIV diagnoses were detected using the Kulldorff’s spatial scan statistic, implemented using SaTScan™ version 9.6 (http://www.satscan.org). Shown in red circles are sub-location clusters of higher new HIV diagnoses with a relative risk of > 1.0 at *p* value< 0.05, and in blue circles sub-location clusters of lower new HIV diagnoses with a relative risk of < 1.0 at *p* value< 0.05. The boundary information for sub-locations in Siaya was obtained as shapefiles from DIVA-GIS (https://www.diva-gis.org/gdata)

HIV testing uptake at sub-location level overlaid with clusters of new HIV diagnoses is shown in Fig. 3. The majority of sub-locations in clusters with higher new HIV diagnoses had high (> 87%) HIV testing uptake, with exceptions observed in sub-locations located south-east of Luhano town; north, north-east, and east of Ngiya town; and west of Ndori town, which all had HIV testing uptake < 82%.

### Associations of new HIV diagnoses in the Bayesian model

In unadjusted analysis, clients aged 20–24 years (uRR 4.44, 95% CI 3.73–5.33), 25–35 years (uRR 8.03, 95% CI 6.84–9.48) and > 35 years (uRR 5.05, 95% CI 4.3–5.96) were more likely diagnosed HIV-positive compared to those aged 15–19 years (Table 3). Men (uRR 0.85, 95% CI 0.79–0.92) were less likely diagnosed HIV-positive compared to women. Compared to clients in monogamous marriage, clients in polygamous marriage (uRR 1.74, 95% CI 1.47–2.04) or separated/divorced (uRR 3.71, 95% CI 3.01–4.51) were more likely diagnosed HIV-positive; while those single (uRR 0.3, 95% CI 0.27–0.34) were less likely diagnosed HIV-positive. Compared to those who reported had tested for HIV 3–12 months ago, those who had never tested (uRR 1.3, 95% CI 1.12–1.5) and those who had tested > 12 months ago (uRR 1.58, 95% CI 1.46–1.71) were more likely diagnosed HIV-positive.

**Table 3:** Factors associated with new HIV diagnoses in non-spatial and spatial models, Siaya County

	Unadjusted relative risk (uRR)	Adjusted relative risk (aRR)			
		Fixed effects only	Spatially unstructured model	Spatially structured model	Convolution spatially unstructured and structured model
	uRR (95% CI)	aRR (95% CI)	aRR (95% CI)	aRR (95% CI)	aRR (95% CI)
Age groups (years)					
15–19	1.00 (ref)	1.00 (ref)	1.00 (ref)	1.00 (ref)	1.00 (ref)
20–24	4.44 (3.73–5.33)	3.55 (2.94–4.31)	3.46 (2.86–4.2)	3.45 (2.85–4.19)	3.45 (2.85–4.2)
25–35	8.03 (6.84–9.48)	4.78 (3.94–5.83)	4.78 (3.93–5.82)	4.76 (3.92–5.81)	4.76 (3.92–5.81)
> 35	5.05 (4.3–5.96)	2.44 (1.99–3)	2.45 (2–3.01)	2.44 (1.99–3)	2.44 (1.99–3)
Sex					
Women	1.00 (ref)	1.00 (ref)	1.00 (ref)	1.00 (ref)	1.00 (ref)
Men	0.85 (0.79–0.92)	0.95 (0.88–1.03)	0.96 (0.89–1.04)	0.96 (0.89–1.04)	0.96 (0.89–1.04)
Marital status					
Married monogamous	1.00 (ref)	1.00 (ref)	1.00 (ref)	1.00 (ref)	1.00 (ref)
Married polygamous	1.74 (1.47–2.04)	1.86 (1.57–2.19)	1.84 (1.55–2.17)	1.84 (1.55–2.17)	1.84 (1.55–2.16)
Separated/divorced	3.71 (3.01–4.51)	3.35 (2.72–4.08)	3.37 (2.73–4.1)	3.36 (2.72–4.08)	3.36 (2.72–4.08)
Single	0.3 (0.27–0.34)	0.49 (0.42–0.55)	0.5 (0.44–0.57)	0.5 (0.44–0.57)	0.5 (0.44–0.57)
Widow/widower	0.98 (0.87–1.1)	1.13 (0.99–1.28)	1.1 (0.97–1.24)	1.1 (0.97–1.24)	1.1 (0.97–1.24)
Time since last HIV test					
< 3 months	1.36 (0.94–1.9)	1.31 (0.9–1.81)	1.31 (0.9–1.83)	1.33 (0.91–1.85)	1.33 (0.91–1.85)
3–12 months	1.00 (ref)	1.00 (ref)	1.00 (ref)	1.00 (ref)	1.00 (ref)
> 12 months	1.58 (1.46–1.71)	1.51 (1.39–1.63)	1.54 (1.41–1.67)	1.53 (1.41–1.66)	1.53 (1.41–1.66)
Never tested	1.3 (1.12–1.5)	2.37 (2.04–2.74)	2.35 (2.02–2.73)	2.35 (2.01–2.72)	2.35 (2.02–2.72)
Sub-location proportion of total HIV-positive clients^a^	1.61 (1.43–1.8)	1.5 (1.34–1.68)	1.49 (1.21–1.82)	1.26 (1.04–1.53)	1.3 (1.07–1.6)
**Random effects**					
Spatially unstructured precision			6.01 (4.37–8.27)		17.39 (9.01–36.14)
Spatially structured precision				2.44 (1.67–3.59)	4.97 (2.5–9.24)
**Model comparison**					
Effective number of parameters		13	125.12	109.09	113.8
Deviation information criterion		11,153.63	10,816.12	10,811.64	10,810.58

^a^The proportion of total HIV-positive clients was defined as the sum of the total new HIV-positive and previously identified HIV-infected clients among those whose eligibility for HIV testing was assessed

Abbreviations: uRR, unadjusted relative risk; aRR, adjusted relative risk; CI, credible interval

The non-spatial and spatial random effect multivariable models used to explore factors associated with HIV-positive diagnosis are shown in Table 3. Of the four multivariable models explored, the convolution model that consisted of both a spatially structured and unstructured random effect model performed best with a deviation information criterion of 10,810.58. In this model, there was no association between sex (men compared to women) and HIV-positive diagnosis. Clients aged 20–24 years (aRR 3.45, 95% CI 2.85–4.20), 25–35 years (aRR 4.76, 95% CI 3.92–5.81) and > 35 years (aRR 2.44, 95% CI 1.99–3.00); clients in polygamous marriage (aRR 1.84, 95% CI 1.55–2.16), or separated/divorced (aRR 3.36, 95% CI 2.72–4.08); and clients never tested (aRR 2.35, 95% CI 2.02–2.72) and those who had tested > 12 months ago (aRR 1.53, 95% CI 1.41–1.66) were more likely to be diagnosed HIV-positive. The proportion of total HIV-positive clients in a sub-location (aRR 1.3, 95% CI 1.07–1.60) was also positively associated with HIV diagnosis. Clients whose marital status was single (aRR 0.50, 95% CI 0.44–0.57) were less likely to be diagnosed HIV-positive.

**Fig. 4:**
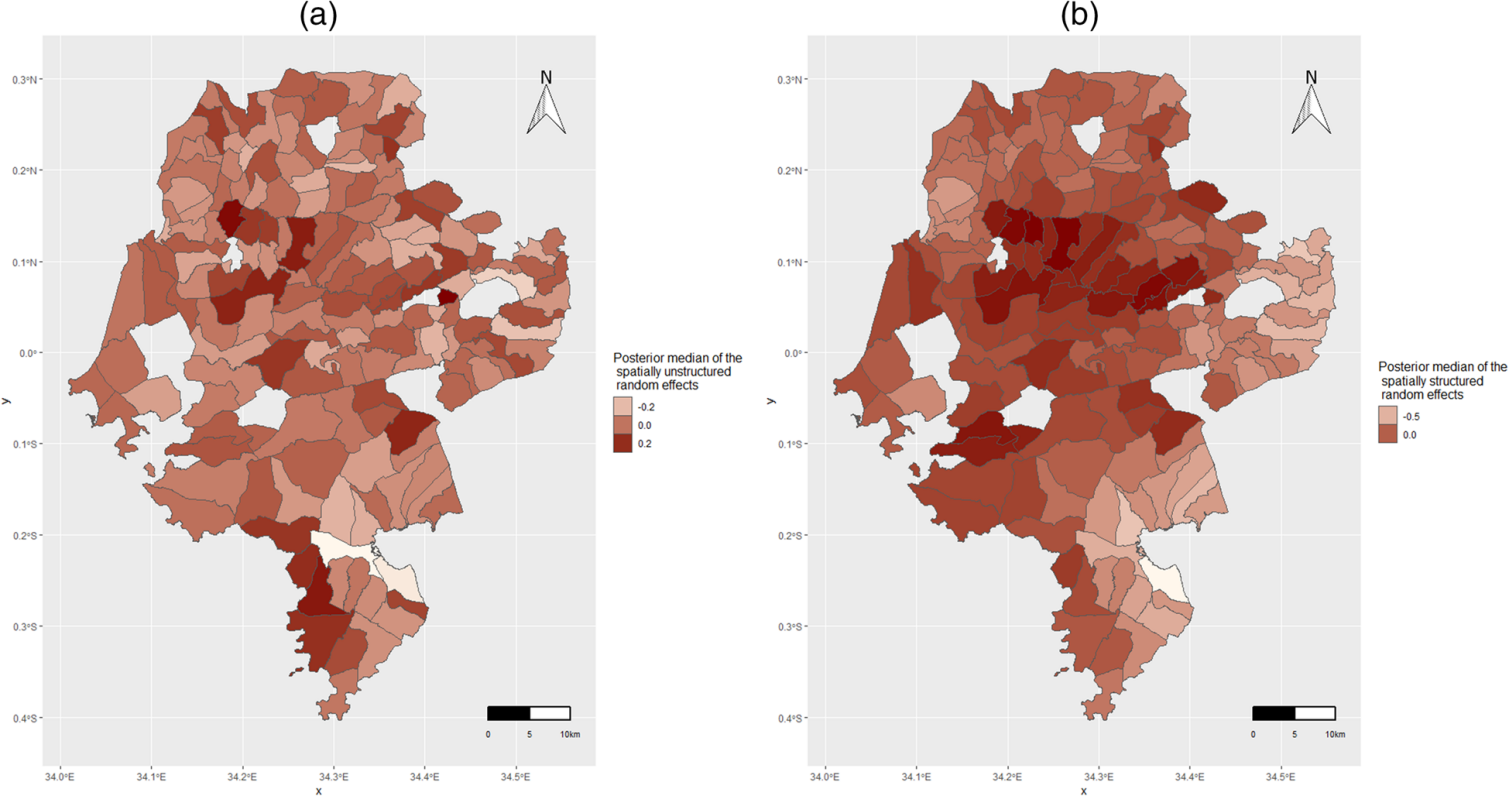
Maps of unstructured and structured random effects of new HIV diagnosis, Siaya County. **a**) Map of estimated median value of unstructured random effects, showing residual variability of new HIV diagnoses when spatial autocorrelation was not taken into account; **b**) Map of estimated median value of structured random effects, showing residual variability of new HIV diagnoses when spatial autocorrelation was accounted for. The maps were generated from the convolution Bayesian Poisson model, and mapped using ggplot2 R package (https://ggplot2.tidyverse.org)

Maps of the unstructured and structured estimated median value of the random effects for each sublocation, generated from the convolution Bayesian Poisson model, are shown in Fig. 4. The maps show the pattern of random effects, that further explain the distribution of new HIV diagnoses, over and above what is explained by the fixed effects (age group, sex, marital status, time since last HIV test and sub-location proportion of total HIV-positive clients). Figure 4 (a) shows the pattern of posterior median unstructured random effects, not taking into account spatial autocorrelation. When spatial autocorrelation was taken into account, as shown in Fig. 4 (b), the pattern of posterior median random effects changed, with more darker areas in the central region, demonstrating higher influence of spatially correlated random effects in this area.

**Fig. 5:**
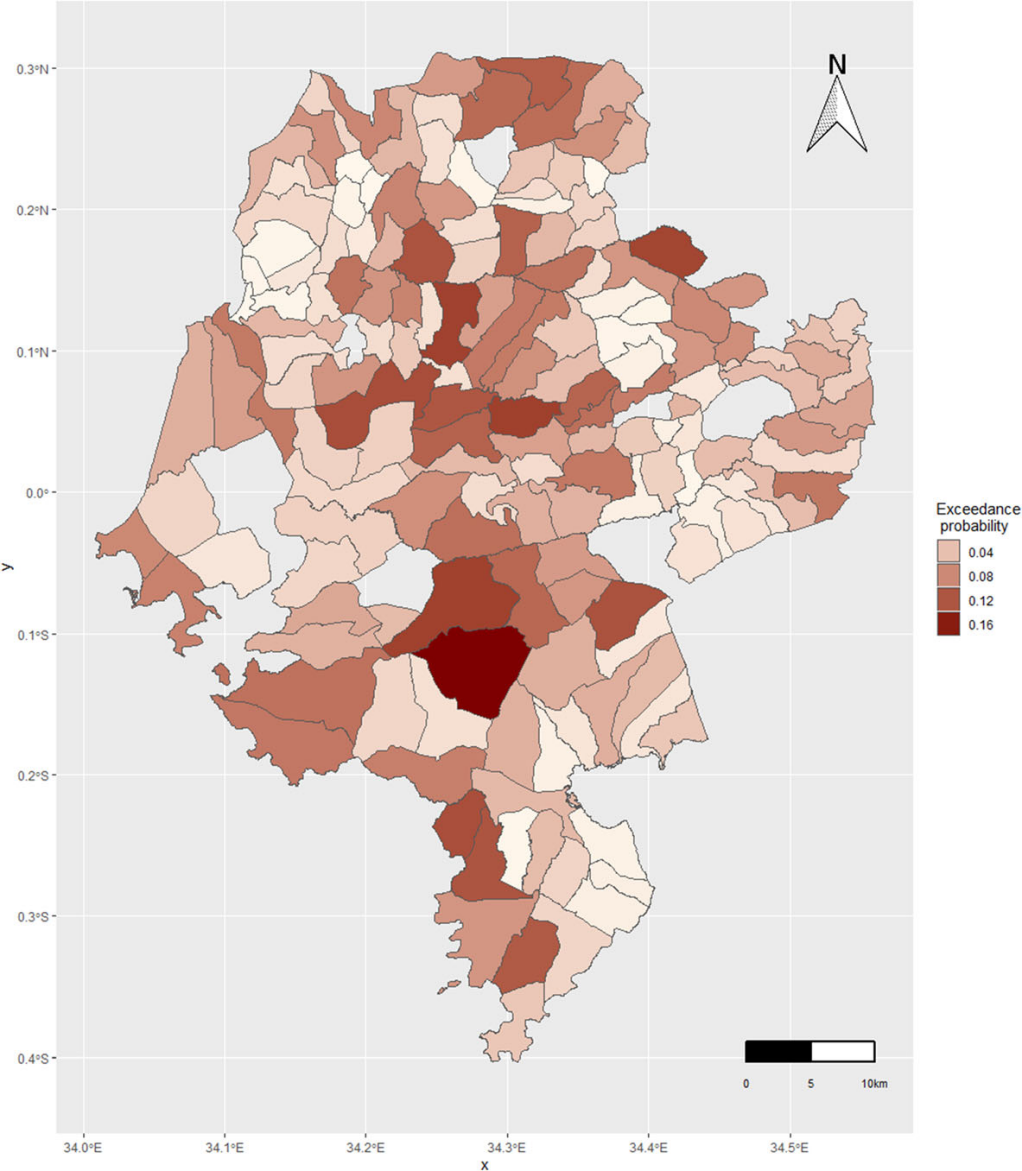
Map of exceedance probability of new HIV diagnosis, Siaya County. The posterior probability of the sub-location’s relative risk to exceed a threshold was calculated using the Bayesian approach. For our analysis, a threshold of 1.25 was used. The darker colors show areas of high probabilities, while the lighter colors show areas of low probabilities. The map was created using ggplot2 R package (https://ggplot2.tidyverse.org)

Sub-location level exceedance probability of new HIV diagnoses is shown in Fig. 5. The darker colors show areas of high probabilities, while the lighter colors show areas of low probabilities.

## Discussion

Our study uniquely demonstrates the use of geospatial analysis in a routine public health program to assess geospatial patterns of new HIV diagnoses, and identify geographic areas where HIV interventions could be targeted with finer granularity. Although the HIV epidemic in Siaya is generalized, our study found spatial variation in new HIV diagnoses, and identified sub-location clusters in which the number of new HIV diagnoses observed was 1.56 to 2.64 times higher than expected. We also identified sub-locations with higher exceedance probability of new HIV diagnoses, indicating areas where the probability of new HIV diagnoses are high. Geographic clusters of higher new HIV diagnoses may be attributed to having a high number of undiagnosed PLHIV, a high number of incident HIV infections, increased access to HIV testing, or a combination of these factors. It would, therefore, be beneficial to target intensified HIV prevention and testing interventions to these areas, as they may have relatively more undiagnosed PLHIV unreached by the program, and continued HIV transmission driven by high viral load levels among undiagnosed and newly diagnosed HIV-infected individuals.

Several studies have described ways to prioritize HIV interventions to specific geographic areas as a means to improve efficiency and cost-effectiveness; these include mapping the geographic distribution of ART coverage [38], and the distribution of sub-populations with higher HIV-risk [11] or higher HIV prevalence [11, 18, 19]. Additionally, studies have described opportunities to utilize geospatial analysis and mapping to support targeting of HIV program interventions towards achievement of HIV epidemic control [20, 39]. To our knowledge, this study is the first to map fine (< 5 km radius) clusters of higher HIV diagnoses using routine data from a home-based HIV testing program. A similar study done in Kenya used routine facility-level HIV testing data to identified facility clusters (at a radius of < 50 km) of newly diagnosed HIV-positive persons across counties with differing HIV burden [21]. Other routinely available HIV testing data [e.g., provider-initiated testing and counseling data at health facilities, data from partner HIV testing services (index testing), antenatal clinic data, etc.] could be used in a similar manner.

Our study further identified sub-locations with both higher new HIV diagnoses and low testing uptake. A study in Zimbabwe demonstrated the use of geospatial analysis to target areas for increased uptake of HIV services, including those with high HIV prevalence [40]. Our findings, therefore, add to the literature base describing the utility of geospatial analysis in identifying areas with potentially high HIV-positive yield that could be efficiently targeted to increase HIV testing uptake.

In Siaya, clusters of higher new HIV diagnoses were found in areas around specific towns, around major roads, near a major road intersection and adjacent to a beach. Although geospatial clustering of new HIV diagnoses has not yet been described in the literature, other studies have described the clustering of higher HIV prevalence [10, 13] and incidence [12] around similar ecological factors. The clustering around ecological features observed in our study suggests that population-level factors related to the ecological features, including socioeconomic, mobility and geographic factors, may influence the clustering of new HIV diagnoses. Surprisingly, however, the sub-location cluster with the highest relative risk was in a predominantly rural area with no prominent ecological features. Furthermore, several sub-locations around towns and major roads had clusters of lower new HIV diagnoses, suggesting that other unidentified factors unrelated to ecological factors, additionally influence the distribution of new HIV diagnoses.

A Bayesian model was used to enable assessment of individual and spatial-level associations of new HIV diagnoses in a spatially-integrated framework. Spatial effects influenced the distribution of new HIV diagnoses, influencing the degree of association of individual-level factors, and further influencing the pattern of random effects (the distribution of new HIV diagnoses not explained by factors in the Bayesian model). In the spatial Bayesian model, we found that clients in polygamous marriage and those separated/divorced were more likely diagnosed HIV-positive, likely due to their higher risk of HIV infection as shown in other studies [41–45]. Polygamous marriages permit concurrent sexual partnerships [46] and correlates with low rates of condom use [47]. Separated/divorced women have been shown to have a higher risk of HIV [48], as these women may seek new sexual relationships that put them at higher risk of HIV, or HIV infection may have contributed to the divorce/separation [49]. Although several studies have documented a correlation between widowhood and higher HIV infection [44, 45, 50], a significant association between widowed individuals and HIV-positive diagnosis was not observed in this study. Similar to findings observed in facility-based testing [51], individuals never tested for HIV, and those tested > 12 months prior, were more likely to be diagnosed HIV-positive. The association between increasing age and higher likelihood of HIV diagnosis found in this study is consistent with higher HIV prevalence observed in older age groups [52–54]. Although other studies have shown that men have lower HIV prevalence compared to women, our spatial model did not find a significant association between HIV-positive diagnosis and sex. The association observed between higher proportion of total HIV-positive clients in a sub-location and higher new HIV diagnoses suggests these areas likely have a relatively high number of undiagnosed PLHIV and ongoing local HIV transmission. Random effects or additional factors beyond those included in the Bayesian model, influenced the distribution of new HIV diagnoses (Fig. 4). This points out to the importance of other factors, likely other individual or population-level factors (including geographic, economic or social), that influenced the pattern of new HIV diagnoses.

Home-based HIV testing conducted in Siaya between May 2016 and July 2017 achieved high (86%) HIV testing uptake among eligible individuals; and was comparable to the testing uptake (64 to 99%) reported in other home-based testing programs in sub-Saharan Africa [55]. The proportion of new HIV diagnoses was low (1.1% HIV-positive yield), slightly lower than that observed in outpatient HIV testing services (1.3% yield) in this setting [51]. The low yield observed is likely due to a diminishing number of undiagnosed PLHIV in the general population, and further highlights the importance of granular spatial analysis to better target HIV testing programs.

We compared the number of individuals aged > 15 years enumerated for home-based testing in the 161 sub-locations included in our analysis (365,798 clients), with 2016/2017 corresponding projected population (435,727 individuals). The projected population was derived using 2009 [56] and 2019 [57] Kenya population census reports. From this, we estimate that majority (~ 84%) of residents aged > 15 years in the 161 sub-locations included in our analysis were enumerated for home-based testing.

Our study had some limitations. First, our results do not represent the whole of Siaya County, as data for 18 sub-locations were excluded; our study did, however, include the majority (90%) of sub-locations in the county. Second, we encountered several limitations owing to the use of routinely collected data for home-based testing, namely: HIV testing procedures were those set for the routine home-based testing program; during enumeration, household residents who reported they would be away for more than one-month following enumeration were excluded, which might have reduced representation of adolescents in boarding schools/colleges; data were not available to verify the number of households in each sub-location enumerated; and variables included in our analysis of factors associated with new HIV diagnoses were limited to those routinely collected, and therefore we were not able to explore other variables likely associated with new HIV diagnoses. Third, per Kenya Ministry of Health guidelines, the assessment of HIV testing eligibility relied on self-reported previous HIV testing, which can be unreliable [58].

Finally, despite literature showing utility of geospatial analysis in informing geographic-targeting of HIV interventions [20, 21, 39], geospatial analysis is not routinely used in public health programs. Our study demonstrates the feasibility of using routine HIV testing data for geospatial analysis, to identify granular (< 5 km) geographic areas to target HIV testing and other interventions. We recommend that countries and programs should integrate geospatial analysis into routine public health program data analysis and use, to inform targeting of interventions to more granular geographic units for maximal epidemiologic impact and efficient resource allocation.

## Conclusions

Our study uniquely demonstrates the use of geospatial analysis in a routine public health program, to identify geographic areas with higher new HIV diagnoses where HIV interventions could be targeted with finer granularity. Additionally, we demonstrate sub-populations with higher HIV-positive yield (i.e., older age groups, those in polygamous marriage or separated divorced, and those never tested for HIV, or tested HIV-negative > 12 months prior), that would benefit from continued targeted HIV testing and prevention interventions. As countries make progress towards HIV epidemic control, integrating geospatial analysis into routine public health programs would help focus interventions to more granular geographic units for maximal epidemiologic impact and efficient resource allocation.

## Supplementary Information

**Additional file 1.**

## Data Availability

The datasets used and/or analyzed during this study are available from the corresponding author upon reasonable request.

## Abbreviations

HIV: Human immunodeficiency virus
TB: Tuberculosis
CDC: Centers for Disease Control and Prevention
CI: Credible interval
ART: Antiretroviral therapy
PLHIV: People living with HIV
AIDS: Acquired Immunodeficiency Syndrome
UNAIDS: Joint United Nations Programme on HIV/AIDS
PEPFAR: President’s Emergency Plan for AIDS Relief
GIS: Geographic Information System
INLA: Integrated Nested Laplace Approximation
uRR: Unadjusted relative risk
aRR: Adjusted relative risk
BYM: Besag-York-Mollié
CAR: conditional autoregressive.

## Footnotes

1 From the manufacturer’s package insert, Determine™ has a sensitivity of 100% and specificity of 99.8%; while First Response® has a sensitivity of 100% and specificity of 99.5%.
