# Supplementary material for: Mapping geographic clusters of new HIV diagnoses to inform granular-level interventions for HIV epidemic control in western Kenya
Source: BMC Public Health. 2021 Oct 23;21:1926. doi: 10.1186/s12889-021-11890-7 (PMC8542332; doi:10.1186/s12889-021-11890-7)

**Supplemental Figures:**

**Figure S1: New HIV-positive yield for clients offered home-based HIV testing in Siaya County.**

d

d

**Figure S2: Sub-location mapping of different characteristics, Siaya County.**


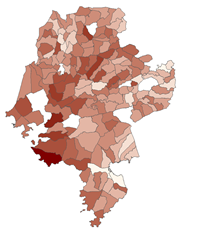

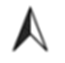

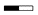


0

5

10 km

**b) Proportion of total HIV-positive clients**

15.0

Proportion of total HIV-positive clients (%)

12.5

10.0

7.5

5.0


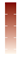

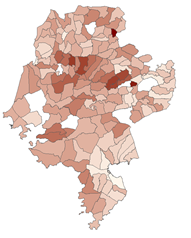


New HIV-positive yield (%)


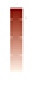


3

2

1


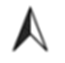

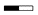


0

5

10 km

**a) New HIV-positive yield**


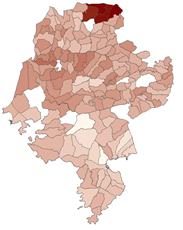


**c) Proportion of clients aged >25 years**

90

80

70

60

50


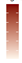


Proportion of clients aged >25 years


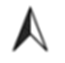


0

5

10 km


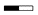

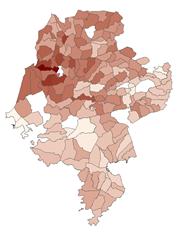


**g) Proportion widowed**


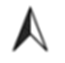


0

5

10 km


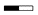


20

15

10

5

Proportion widowed (%)


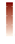

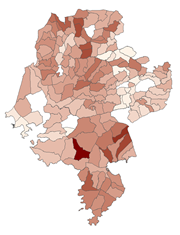


**h) Proportion never tested for HIV**


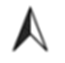


0

5

10 km


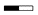


15

10

5

Proportion never tested for HIV (%)


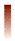

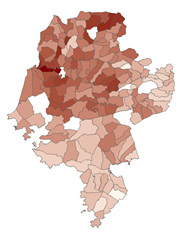


**d) Proportion women**


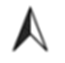


0

5

10 km


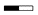


Proportion women (%)

65

60

55

50


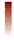

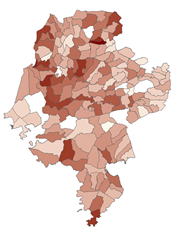


**e) Proportion married polygamous**

5

4

3

2

1

Proportion married polygamous (%)


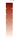

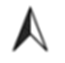


0

5

10 km


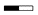

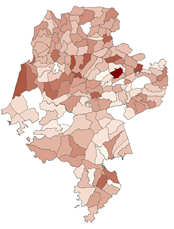


**f) Proportion separated/divorced**


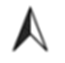


0

5

10 km


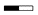


2.5

2.0

1.5

1.0

0.5

Proportion separated/divorced (%)


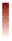

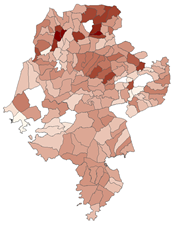


**i) Proportion tested for HIV >12 months ago**


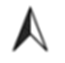


0

5

10 km


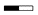


50

40

30

20

10

Proportion tested for HIV >12 months ago (%)


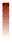

Supplement: Supplementary file 1 — Additional file 1. [file 12889_2021_11890_MOESM1_ESM.docx]
